# Formulation and Characterization of a Novel Oxidized Alginate-Gelatin-Silk Fibroin Bioink with the Aim of Skin Regeneration

**DOI:** 10.61186/ibj.27.5.280

**Published:** 2023-08-23

**Authors:** Khadijeh Sanaei, Ali Zamanian, Shohreh Mashayekhan, Tayebe Ramezani

**Affiliations:** 1 Department of Nanotechnology and Advanced Materials, Materials and Energy Research Center, Karaj, Iran;; 2 Department of Chemical and Petroleum Engineering, Sharif University of Technology, Tehran, Iran;; 3 Faculty of biological sciences, Kharazmi University, Tehran, Iran

**Keywords:** Alginate, Bioprinting, Fibroin, Gelatin

## Abstract

**Background:**

In the present study, a novel bioink was suggested based on the OAlg, GL, and SF hydrogels.

**Methods:**

The composition of the bioink was optimized by the rheological and printability measurements, and the extrusion-based 3D bioprinting process was performed by applying the optimum OAlg-based bioink.

**Results:**

The results demonstrated that the viscosity of bioink was continuously decreased by increasing the SF/GL ratio, and the bioink displayed a maximum achievable printability (92 ± 2%) at 2% (w/v) of SF and 4% (w/v) of GL. Moreover, the cellular behavior of the scaffolds investigated by MTT assay and live/dead staining confirmed the biocompatibility of the prepared bioink.

**Conclusion:**

The bioprinted OAlg-GL-SF scaffold could have the potential for using in skin tissue engineering applications, which needs further exploration.

## INTRODUCTION

Three-dimensional bioprinting has drawn considerable attention in TE applications and medicine reconstruction as a novel technique^[^^1^^,^^2^^]^. To achieve successful bioprinting with minimal process-induced cell damage, preparation of well-formulated bioinks is necessary. Bioinks are natural or synthesized hydrogels that can be laden by cells or other macromolecules^[^^3^^,^^4^^]^. Alg, as a natural polysaccharide, is a favorable hydrogel for bioink formulations, because of its biomimicry to the natural extracellular matrix, viscoelasticity, biocompatibility, biodegradability, and quick gelation^[^^5^^-^^9^^]^. However, the employment of Alg as a bioink faces some challenges. For instance, Alg is inherently unable to support cell attachment and proliferation due to the lack of cell-interactive motifs. Additionally, the biodegradability of Alg is relatively low owing to the absence of Alg-degrading enzymes in the human body. To solve these problems, scientists have suggested chemical modifications of Alg. It is well known that OAlg or Alg di-aldehyde provides a faster degradation rate compared to the conventional Alg^[^^5^^,^^10^^-^^12^^]^. Moreover, this chemical modification provides some aldehyde groups on the Alg structure that enables OAlg to spontaneously crosslink with proteins through Schiff base reactions^[^^13^^,^^14^^]^. 

In recent years, the combination of OAlg with proteins such as GL, SF, or both to form self-crosslinking bioinks has been studied^[^^11^^,^^15^^,^^16^^]^. GL is a water-soluble, bio-compatible polymer containing Arg-Gly-Asp sequence that improves cell adhesion^[^^17^^,^^18^^]^. SF is a natural polymer with unique features such as biocompatibility, biodegradability, high mechanical, and high cell-supporting properties^[^^19^^]^. While the unique properties of OAlg, GL, and SF hydrogels have been investigated before, designing an ideal bioink that can support long-term cell survival in 3D bioprinting process remains challenging.

In this study, we prepared a ternary cell-laden bioink, comprising OAlg, GL, and SF, with the aim of long-term cell viability evaluation. After finding the optimum bioink formulation, a 3D bioprinting procedure was performed, and the printed scaffolds were tested with different in vitro analyses. The optimum sample was proposed for skin TE applications. Herein, the 3D fabricated scaffolds were crosslinked via a dual-stage crosslinking process; first, a covalent crosslinking was formed according to Schiff base reaction between the aldehyde groups of OAlg and amine groups of proteins (GL and SF) before the 3D bioprinting process, and second, ionic crosslinking occurred by the addition of calcium chloride after the 3D bioprinting process. To the best of our knowledge, a bioink that combines all the above-mentioned biomaterials to fabricate a cell-laden 3D scaffold has not yet been developed.

## MATERIALS AND METHODS

**Materials**

Silkworm cocoons (*Bombyx mori*) were purchased from a local market in Iran. Mouse fibroblast L929 cells were provided from MERC (Iran). DMEM, fetal bovine serum, and penicillin-streptomycin were acquired from (BIO-IDEA, Iran). Alginic acid sodium salt (Mw of 120-190 kDa), GL powder (G2500; type A from porcine skin), sodium periodate, ethylene glycol, sodium chloride, dialysis tube (D-0405), lithium bromide, sodium carbonate, calcium chloride, MTT, AO, PI, and dimethyl sulfoxide were all purchased from Sigma-Aldrich (USA). Ninhydrin and sodium citrate were provided from Merck (Germany). 

**Synthesis of OAlg**

OAlg was synthesized as described before^[^^13^^]^. Briefly, an aqueous solution of Alg (2.5% w/v) was prepared and mixed slowly with sodium periodate solution (1.35 w/v; 60 mL) in the dark at RT. After 24 h, the ethylene glycol (21 µL) was added to quench the reaction via stirring for 30 min. Next, sodium chloride was added to the solution, followed by adding ethanol for precipitation of OAlg. The precipitates were collected by centrifugation and dissolved in DW. The washing steps were repeated three times and the purified OAlg was lyophilized and kept at 4 °C. The oxidation degree was measured by UV/vis spectroscopy as described formerly^[^^20^^]^.

**Synthesis of SF**

The SF protein was processed as previously reported^[^^21^^]^. In brief, small pieces of cocoons (5 g) were added to an aqueous solution of Na_2_CO_3_ (0.02 M) at 98 ± 1 °C under stirred conditions for 30 min. Then SF was removed and rinsed three times by DW for 20 min. After being air-dried for 24 h, the SF was dissolved in 9.3 M lithium bromide solution at 60 ±1 °C for 4 h. The SF solution was dialyzed by applying a dialysis bag (12 kDa) and placed in DW at RT for three days. The dialysis water was renewed several times to speed up the removal of lithium bromide. In the end, the SF solution was stored at a refrigerated temperature. To determine the concentration of SF, 1 mL of solution was weighed before and after drying at 60 ± 1 °C for 24 h.

**Preparation of bioink**

The preparation of bioink, composing of OAlg, SF, and GL, was carried out according to a previously mentioned method^[^^22^^]^. Briefly, OAlg and GL were respectively dissolved in DW and DMEM at 37 ºC and then mixed to form an utterly homogeneous solution. Finally, the purified SF solution was added to the OAlg-GL solution and vortexed slowly at RT^[^^23^^]^. Hydrogels at a concentration higher than 10 mg/mL showed poor cell viability, and at low concentrations, hydrogels exhibited poor structural integrity^[^^24^^]^. Thus, 4% (w/v) concentration of OAlg and varied concentrations of GL and SF from 0% (w/v) to 6% (w/v) were used. The total concentration of bioink was kept constant at 10% (w/v). The seven prepared bioink formulations are summarized in Table 1.

**Table 1:** Composition of the OAlg-based bioink

Code	OAlg (%w/v)	GL (%w/v)	SF (%w/v)	Total concentration (%w/v)
**G6S0**	4	6	0	10
**G5S1**	4	5	1	10
**G4S2**	4	4	2	10
**G3S3**	4	3	3	10
**G2S4**	4	2	4	10
**G1S5**	4	1	5	10
**G0S6**	4	0	6	10

**Preparation of cell-laden bioink**

Mouse fibroblast (L929) cells were cultured in DMEM supplemented with 10% (v/v) fetal bovine serum and 1% (v/v) penicillin-streptomycin in 5% CO_2 _at 37 °C. Upon reaching suitable confluency, the culture flasks were harvested. The cells were detached from the floor of culture flasks, counted, and loaded to bioink to prepare a cell-laden bioink containing 2 × 10^6^ cells/mL^[^^25^^]^.

**Three-dimensional bioprinting process**

The 3D bioprinting process was carried out by using a dual-nozzle extrusion-based 3D bioprinter machine (BioFabX2, Omid Afarianan Co., Iran), which was equipped with three-axis motion sensor, a temperature controller (up to 220 ºC) and a pneumatic controller system (<750 kPa). The bioinks were printed into 3D grids (20 × 20 mm^2^; 10 layers) and designed using SolidWorks 2020 (version 28). The printing speed and pressure were kept constant at 4 mm/s and 30 kPa, respectively, and the samples were printed via a 25-gauge needle (ID 260 mm). Both the ejection chamber and bed temperature were set at RT during the extrusion process. After finishing the printing process, the samples were crosslinked by CaCl_2 _(5% w/v) for 60 min, and excess CaCl_2 _was discarded by washing three times with PBS^[^^26^^]^.

**Characterization of bioink**

***HNMR***

To confirm the OAlg synthesis, an HNMR test was carried out before and after the oxidation modification using a spectrophotometer (Varian INOVA 500 MHz, Varian, USA) in D_2_O water at 60 °C^[^^27^^]^. 

***GPC***

In order to determine the Mw of Alg and OAlg, the GPC analysis was used by applying an Agilent 1100 GPC device (Agilent technologies, USA). The polyethylene glycol/oxide standards were employed for calibration^[^^28^^]^. 

***ATR-FTIR***

The ATR-FTIR spectroscopy was performed using a Thermo Nicolet Avatar spectrometer (Thermo Nicolet Corporation, USA) to investigate the chemical groups of the samples. All spectra were measured in the range of 600 to 4000 (cm^-1^) with an average of 64 scans at RT^[^^29^^]^.

***Rheological properties***

To determine the rheological behavior of bioinks, frequency sweep test, shear rate sweep test, and recovery test were evaluated by applying a cone-plate rheometer (Anton Paar, Austria) in the oscillatory mode (diameter of 50 mm and 2° cone angle) at RT. The frequency sweep test was performed to assess the storage modulus (G') and the loss modulus (Gʺ) from 0.1 to 100 Hz at 2% strain^[^^14^^]^. The recovery test was carried out to evaluate the recovery behavior of the bioinks in three steps: the shear rates of 0.1 s^-1^ for 60 s^-1^ (step 1), 100 s^-1^ for 10 s (step 2), and 0.1 s^-1^ for 60 s (step 3)^[^^26^^,^^30^^]^. 

***Printing accuracy***

To evaluate the printing accuracy, three samples were printed, and images were captured with a camera (12 megapixels, and 1.4 μm pixel size). The ImageJ software version 2.0 (NIH, USA) was applied to determine the image dimensions and threshold. The printing accuracy (%) was measured using Equation 1^[^^23^^]^ wherein Ai (X mm^2^) and A (64 mm^2^) show the printed and designed area for each sample, respectively.

$\mathrm{Printing}\mathrm{accuracy}\left(\%\right)=\left[1-\frac{\left|A_{i}-A\right|}{A}\right]\times 100$

(1)

***Degree of crosslinking***

The ninhydrin staining was employed to determine the percentage of free amines. For this purpose, ninhydrin solution with a concentration of 2% (w/v) was prepared. Then 0.5 mg of the crosslinked samples were added to ninhydrin solution. Non-crosslinked samples were considered as the control sample. Afterward, the mixture was heated at 100 °C for 20 minutes. Next, the absorption of the solution was read at a wavelength of 570 nm using a UV-vis spectrophotometer (WPA Biowave II, Biochrom Cambridge, UK). The degree of crosslinking (%) was obtained using Equation 2^[^^31^^]^ wherein, A_c_ and A_u_ express the absorption of crosslinked and non-crosslinked scaffolds, respectively.

$\mathrm{Degree}\mathrm{of}\mathrm{crosslinking}(\%)=\left(1-\left(\frac{A_{c}}{A_{u}}\right)\right)\times 100$

(2)

***Gelation time***

To determine the gelation time, 10 mL of hydrogels were prepared in a 15-mL breaker and stirred at 250 rpm and 37 °C using a 10-mm magnet. The gelation time was recorded after SF/GL addition to OAlg solution until complete gelation, i.e. the magnet could no longer rotate^[^^32^^]^. 

**Characterization of scaffolds**

***Porosity test***

The porosity calculation of each scaffold was assessed using a liquid displacement method, in which a graduated cylinder was filled with a certain volume (V_1_) of ethanol. The freeze-dried samples were immersed in ethanol, followed by conducting repeated vacuum-ization until no bubbles were observed. The total volume of ethanol containing the saturated scaffold was noted as V_2_, and the volume of the residual ethanol after the removal of the saturated scaffold was recorded as V_3_. The porosity of the scaffolds (P) was obtained by Equation 3^[^^33^^]^.

$P\left(\%\right)=\left[\frac{\left(V_{1}-V_{3}\right)}{\left(V_{2}-V_{3}\right)}\right]\times 100$

(3)

***Wettability***

The wettability of the scaffolds was investigated by the sessile-drop contact angle test. For this purpose, a CAM device (MERC) was applied, and images were taken using a Color Industrial Camera (DFK 23U618-USB-3.0, Imaging Source Co., USA). For each test, 4 µLof DW was slowly dropped on the surface of the specimen in three different parts, and the results were reported.

***Swelling behavior***

The swelling properties were performed according to a conventional gravimetric method. In brief, the 3D printed constructs were weighted and soaked in PBS (pH 7.4) at 37 °C. At certain times (1, 3, 5, 7, 12, and 24 h), the swelled scaffolds were brought out from the PBS, and the excess water on the surface was discarded by a filter paper and then weighed. The experiments were performed with three replications, and the swelling ratio ($Q_{m}$) was expressed as Equation 4^[^^16^^,^^34^^]^, where $W_{d}$ and $W_{s}$ demonstrate the initial and final weight of the specimens, respectively.

$Q_{m}\left(\%\right)=\frac{W_{s}-W_{d}}{W_{d}}\times 100$

(4)

***Degradation rate***

The pre-weighed samples were incubated in PBS at 37 °C for 3, 7, 14, 21, and 28 days. The mass remaining (%) was obtained from equations 5 and 6^[34]^ wherein $W_{i}$ and $W_{d}$ are the dry weight of scaffolds before and after immersing in PBS solution, respectively.

% Mass degraded MD=Wi-WdWd×100

 (5)

\% Mass remaining = (100 − MD) 

 (6)

***Mechanical behaviors***

The mechanical behaviors of the samples were investigated using a tensile testing machine (Hounsfield H10-Ks, USA). Briefly, samples with the dimensions of 40 × 15 × 3 mm^3^ were stretched at the strain rate of 2 mm.min^-1^ until the fracture point. For each sample, a stress-strain curve was attained, and the elastic modulus (E), ultimate strength, and elongation (%) at the breaking limit were reported^[^^35^^]^. 

***MTT assay***

The biocompatibility of cell-laden scaffolds was examined using MTT analysis based on the ISO 10993-5 standard. First, specimens with the constant volume of 200 µLwere immersed in 48-well culture plates. Next, a culture medium (100 μL/wells) was added, and the plates were transferred to a cell culture CO_2_ incubator at 37 °C. At different time intervals (1, 3, and 7 days), the culture medium was discarded, and sodium citrate solution (3.2% w/v) was added to each well and then removed after 10 min^[^^36^^]^. Afterward, MTT solution (0.5 mg/mL in PBS) was added to each well and incubated. After 4 h, the solution was discarded, and dimethyl sulfoxide (100 µL) was added to each well. Finally, the absorption was measured using an ELISA microplate reader (DANA, model-DA3200, GARNI Medical Engineering Co., Iran) at 490 nm. Two-dimensional culture was considered as the control.

***Live/dead staining***

AO/PI staining was applied to recognize live and dead fibroblast cells embedded in the 3D printed construct, as mentioned previously^[^^37^^]^. Briefly, after 1, 7, 14, and 21 days of cell encapsulation, all the media inside the wells were discarded, and the samples were precisely washed with PBS several times. Then the samples were treated with AO (0.67 × 10^-3^ mM) and PI (75 × 10^-3^ mM) solution (1:1) and kept in the dark for 15 min. After careful washing the samples with PBS, fluorescence images were taken by a fluorescent Microscope (Labomed T121100, USA).

**Statistical analysis**

All experiments were repeated at least three times, and the results were reported as the mean ± standard deviation (STDEV). For all quantitative assays, statistical analyses of data were carried out by using a one-way ANOVA analysis, followed by Tukey's tests by applying an SPSS (V. 16.0) software. The p value less than 0.5 was considered as statistically significant.

## RESULTS AND DISCUSSION

**Characterization of bioink**

***HNMR, GPC, and ATR-FTIR analysis***

**Fig. 1:**
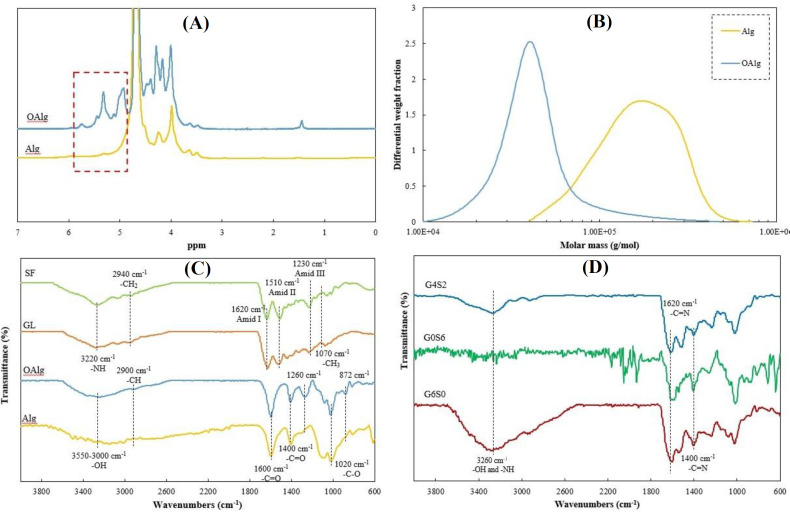
(A) HNMR and (B) GPC spectra of Alg and OAlg. The ATR-FTIR spectra of (C) initial materials (Alg, OAlg, GL, and SF) and (D) final prepared bioinks (G6S0, G0S6, and G4S2)

The HNMR spectra of Alg before and after oxidation procedure are shown in Figure 1A. As can be seen in this Figure, the sharp peaks at 3.6-4.9 ppm were corresponded to the  guluronic (G) and mannuronic (M) acid units of Alg. However, the oxidation led to the appearance of a new peak at around 4.2 ppm, which was related to the proton of the oxidized G unit^[^^28^^]^. Furthermore, the appeared peaks at 5.15-5.75 ppm can be related to the formation of hemiacetal groups^[^^38^^]^. The GPC results of Alg and OAlg are shown in Figure 1B. The results revealed that non-oxidized Alg had approximately 182.0 kg/mol Mw, while after the chemical modification of Alg, the Mw reduced to 47.8 kg/mol in OAlg. Although the reduction of Mw decreases the viscosity of Alg^[^^39^^]^, it will be favorable for biomedical applications due to facile excretion of low Mw Alg (M_w_<80 kg/mol) from the human body^[^^20^^]^. The ATR-FTIR spectra of initial materials (Alg, OAlg, GL, and SF) and the prepared bioinks (G6S0, G0S6, and G4S2) are illustrated in Figures 1C and 1D, respectively. In the spectrum of Alg, the broad peak at 3000-3550 cm^-1^ was related to hydroxyl (OH^-^) groups. Additionally, two sharp peaks were detected at 1600 cm^-1^ and 1400 cm^-1^, which were represented the carboxyl groups (COO ) of Alg. Moreover, a characteristic peak was discovered at 1020 cm^-1^ related to the C-O absorption peak^[^^11^^,^^40^^]^. In the spectrum of OAlg, the COO^-^ peaks were still present; however, a slight shift was observed. Moreover, the intensity of OH^-1^ stretching mode decreased, and the band at 802 cm^-1^ )symmetrical cyclic ethers (C-O-C)( changed^[^^14^^]^. In addition, the aldehyde groups (CHO)-related peak, which is usually detected in the peaks 1725-1751 cm^-1^, was not recognized due to the formation of hemiacetal configuration among free aldehyde groups with adjacent hydroxyl groups^[^^10^^,^^15^^]^. The peak appeared at around 872 cm^-1 ^was related to the hemiacetal groups of OAlg^[^^41^^]^, and a new peak was detected at about 2900 cm^-1^, which was attributed to the C-H bond in the aldehyde groups^[^^15^^]^. These results indicated the effective cleavage of Alg chains and the successful synthesis of OAlg. In the ATR-FTIR spectra of the GL and SF, the absorption bands of amide I (1620 cm^-1^), amide II (1510 cm^-1^), and amide III (1230 cm^-1^) were observed. Furthermore, the broad hydroxyl peak was detected in both GL and SF spectra^[^^13^^,^^34^^]^. As seen in the spectrum of G6S0, the peak of amide I shifted to 1600 cm^-1[^^34^^]^. Additionally, the range of hydroxyl groups (3260 cm^-1^) in the G6S0 bioink was broader compared to the spectra of the OAlg and GL. Moreover, the peaks at 1400 cm^-1^ and 1630 cm^-1^ demonstrated the C=N bands in G6S0 hydrogel. Similar to G6S0, the G0S6 spectra displayed the absorption peaks at around 1400 cm^-1^ and 1620 cm^-1^ due to the presence of C=N bonds. In the spectrum of G4S2, sharp peaks at 1620 cm^-1^ confirmed the Schiff base bond (C=N) formation^[^^16^^]^.

**Fig. 2:**
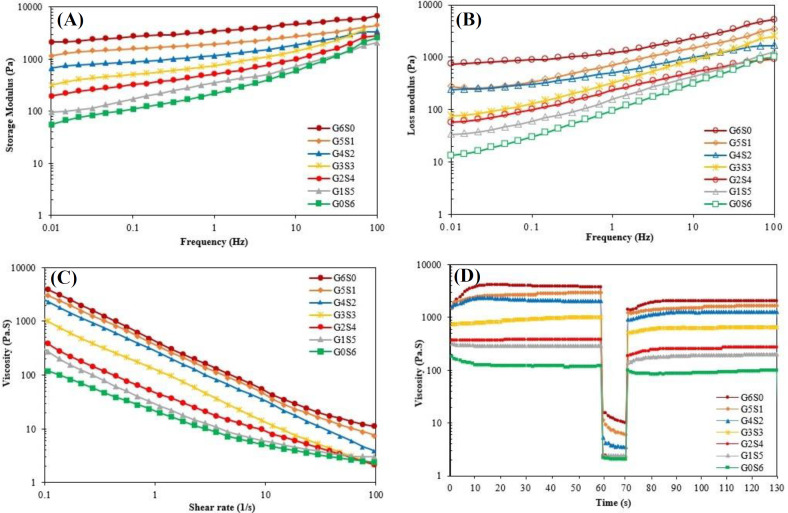
Viscosity and rheological properties of bioinks (G6S0, G5S1, G4S2, G3S3, G2S4, G1S5, and G0S6). (A) Storage modulus (G’) and (B) loss modulus (G’’) as a function of angular frequency (Hz); (C) viscosity as a function of shear rate (0.1-100 s^-1^); (D) viscosity as a function of time (the recovery behavior)

***Rheological properties***

The bioink of high viscosity level and the proper ratio of Gʹ to Gʺ shows improved printability due to increased mechanical strength, which causes sustaining structural integrity^[^^42^^,^^43^^]^. In contrast, the bioink of low viscosity level is favorable for cell encapsulation because it reduces the required printing pressure, resulting in lower shear stress to cells^[^^44^^,^^45^^]^. The rheological properties of OAlg-based bioinks were characterized by frequency sweep, shear rate sweep, and recovery tests, and the results are shown in Figure 2. According to Figure 2A and 2B, it was observed that both Gʹ and Gʺ gradually decreased by increasing SF/GL ratio. In this regard, the Gʹ and Gʺ of G6S0 were obtained as 3400.0 Pa and 1225.0 Pa, while the Gʹ and Gʺ of G0S6 were measured as 217.8 Pa and 95.6 Pa, respectively. The higher Gʹ, compared to the Gʺ, can be an indicator of the gelation state of all tested bioinks^[^^46^^]^. The shear rate sweep test was performed on the OAlg-based bioinks, and the viscosity of the bioinks as a function of shear rate (0.1-100 s^-1^) are shown in Figure 2C. It was observed that all hydrogels exhibited shear-thinning behavior due to viscosity reduction as the shear rate increased^[^^1^^,^^47^^]^. Accordingly, at all shear rates, the viscosity of hydrogels was decreased by increasing the weight ratio of SF to GL. It is well known that the viscosity was increased by adding GL and SF to OAlg, due to crosslinking interactions, based on Schiff base reaction and the formation of a C=N bond among aldehyde groups of OAlg and free amine groups of proteins^[^^11^^,^^48^^]^. Therefore, it is assumed that GL increases the hydrogel strength more than SL and creates intermolecular interactions with OAlg chains. The recovery test is shown in Figure 2D in three shear rate stages (0.1, 100, and 0.1 s^-1^). As can be seen in the Figure, the viscosity recovery (%) increased by elevating the weight ratio of SF to GL in all bioinks, whereas the recovery capability of G6S0, G5S1, G4S2, G3S3, G2S4, G1S5, and G0S6 bioinks were achieved 54%, 55%, 62%, 63%, 66%, 67%, and 71%, respectively. As a result, the prepared bioinks showed shear-thinning and thixotropic properties and can be used as a suitable bioink for 3D printing based on the investigation of the rheological properties.

***Printability,*** ***degree of crosslinking, and gelation time***

The printability measurements of the OAlg-based bioinks are summarized in Table 2. Generally, the hydrogels tend to spread after printing due to gravity, tension, surface energy, and the weight of the top printed layers^[^^44^^,^^48^^]^. Therefore, the measured area of printed constructs was higher than that of the designed model in all cases. As shown in Table 2, the printing accuracy of G6S0 was very low (24 ± 5%). However, the width of the strand became smoother by increasing SF concentration from 0% to 2% (w/v) and decreasing GL concentration from 6% to 4% (w/v). G4S2 bioink showed the appropriate printing accuracy (92 ± 2%). However, the printing accuracy was decreased by a further increase of SF/GL ratio; hence, the printing accuracy of the G0S6 sample was measured at approximately 71 ± 3%. Moreover, the addition of 2% (w/v) SF led to the optimum the optimum rheological results due to the interactions of polymer chains, such as crosslinking and chains locking together, and even facilitating chains movements^[^^44^^]^. The viscosity of bioink continuously reduced by increasing the SF/GL ratio, while a maximum printing accuracy occurred at the appropriate ratio of the SF to the GL. Therefore, G4S2 sample was selected as an optimum bioink composition based on the rheological and printability studies. The degree of crosslinking (%) and gelation time of the prepared scaffolds were measured as shown in Table 2. The degree of crosslinking was calculated based on the ninhydrin colorimetric test and was determined as 42.41 ± 0.9%, 41.32 ± 1.1%, and 38.24 ± 1.1% for G6S0, G4S2, and G0S6 bioinks, respectively. There are various effective factors in the crosslinking of OAlg-based hydrogels, including the amount of OAlg and GL, the deformation degree of polymer chains, the oxidation degree, Mw, and the length of the OAlg polymer chain^[^^16^^]^. While the degree of crosslinking is effective in the stability of the final prepared scaffolds in terms of mechanical stiffness and shape fidelity, extra crosslinking has negative effects on fluidic, nutrition, and oxygen exchange capability, swelling ratio, biodegradability, and as a result cell viability^[^^49^^]^. Therefore, the degree of crosslinking should be modulated based on the required properties. According to the results, gelation time was measured as 1589 ± 84, 1601 ± 97, and 1760 ± 73 s for G6S0, G4S2, and G0S6 scaffolds, respectively. Results showed that all the gelation times were in the appropriate range for clinical applications^[^^37^^]^.

**Table 2:** Morphological observation, printing accuracy (%), and physicochemical properties of bioinks

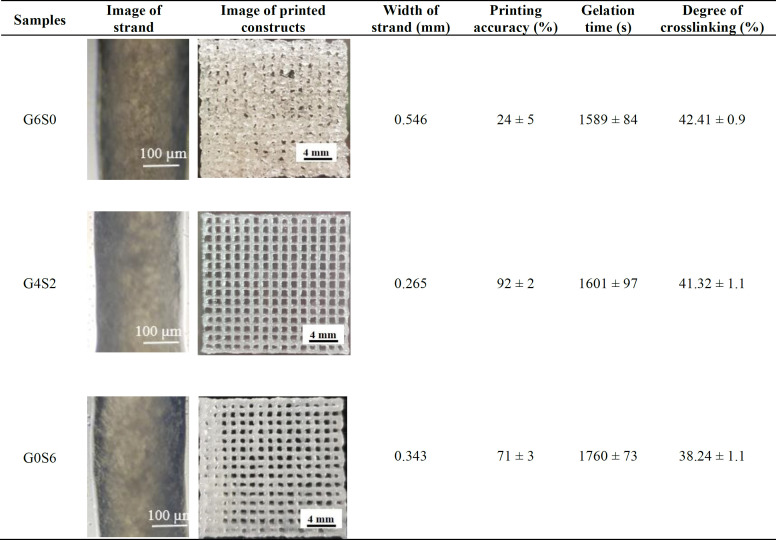

**Characterization of scaffold**

***Porosity analysis, wettability, swelling behavior, and degradation rate***

**Fig. 3:**
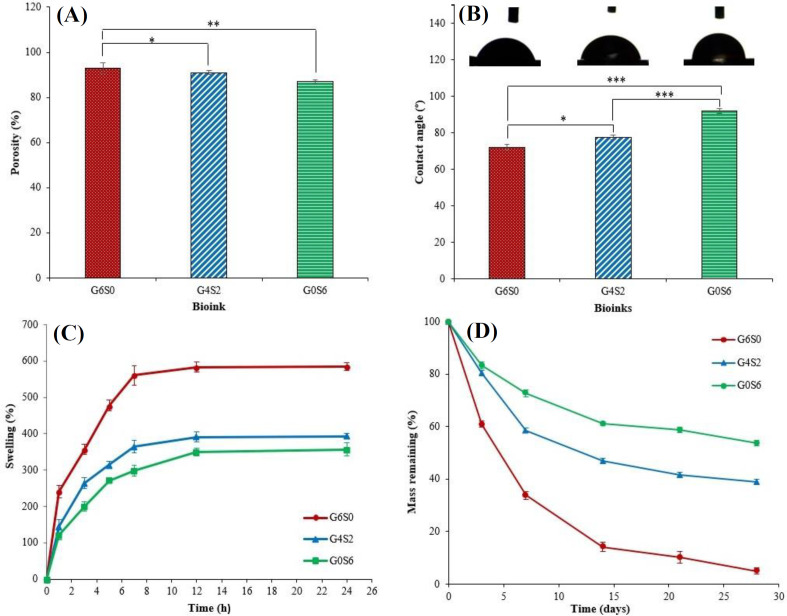
(A) Percentage of porosity, (B) contact angle results, (C) swelling capacity, and (D) degradation ratio of G6S0, G4S2, and G0S6 scaffolds

As shown in Figure 3A, the porosity percentage of the G6S0, G4S2, and G0S6 scaffolds were calculated as 93 ± 2%, 91 ± 2%, and 86 ± 3%, respectively. For the purpose of skin TE, the percentage of porosity should be more than 90% in order to supply oxygen and nutrients and remove wastes^[^^50^^]^. Thus, the optimized sample selected in this research (G4S2) has the appropriate percentage of porosity to support the skin cells during the healing process. Hydrophilic surfaces are generally preferred due to higher water, protein, and cell absorption in the physiological environment and facilitate cell interactions^[^^51^^]^. Also, one of the main functions of the skin is that it acts as a barrier to the surrounding environment. It has been reported that the contact angle of the natural human skin varies between 57° to 92° to play a barrier role, depending on the amount of sebum^[^^52^^,^^53^^]^. Herein, the results of contact angle measurements are shown in Figure 3B. As can be seen in the Figure, the measured contact angles of G6S0, G4S2, and G0S6 scaffolds were found to be 72 ± 1.5°, 76 ± 1.3°, and 92 ± 1.3°, respectively, indicating the hydrophilicity of G6S0 and G4S2 scaffolds and the hydrophobicity of G0S6 scaffold. Thus, it can be concluded that the hydrophilicity decreases with increasing the concentration of SF and decreasing the concentration of GL. Therefore, G4S2 sample was in the ideal range of wettability for both cell adhesion and barrier role. The swelling capacity of the scaffold affects the fluid, nutrition, and oxygen exchange capability. Therefore, it is known as a vital parameter in the skin TE scaffolds, especially in the early stages of wound healing^[^^33^^,^^54^^]^. When an ionically crosslinked Alg scaffold is placed in the body environment, calcium ions are exchanged with sodium ions of body fluid^[^^55^^]^. This exchange disturbs the charge balance of the Alg scaffold and creates electrostatic repulsion between carboxyl ions and negative charge, therefore leading to scaffold swelling and water absorption^[^^29^^]^. Herein, the swelling capacity of the G6S0, G4S2, and G0S6 scaffolds was evaluated using soaking in PBS solution, and the results are illustrated in Figure 3C. As can be seen in the Figure, all the samples showed a high amount of water absorption capacity, and the swelling ratio was evaluated to be 580% ± 15.70, 394% ± 7.45, and 346% ± 18.34 for G6S0, G4S2, and G0S6 bioinks after 24 hours, respectively. The results revealed that the swelling ratio decreased with the addition of SF and the reduction of GL concentrations. In general, the higher compact polymer network with higher crosslinked volume decreases the swelling ratio^[^^37^^,^^54^^,^^56^^]^. It has been reported that the lower concentration of GL leads to a higher tendency to silk II (β-sheets) formation, which is more hydrophobic than silk I (α-helix)^[^^57^^]^. Moreover, SF consists of many hydrophilic groups, such as OH^-^, NH_2_, and COO^-^, which can increase the swelling ratio by absorption of water molecules in the physiological environment. On the other hand, these hydrophilic functional groups can crosslink with Ca^2+^ ions to form β-sheets^[^^58^^]^. Therefore, it can be concluded that by increasing the weight ratio of SF to GL, the formed polymer network will be more compact with hydrophobic groups, which leads to the decreased swelling capacity. Figure 3D shows the degradation rate of the G6S0, G4S2, and G0S6 scaffolds after 3, 7, 14, 21, and 28 days of immersion in PBS solution. One of the main goals of the oxidation modification of Alg and synthesis of OAlg was to adjust the degradation rate for tissue regeneration and t be eliminated from the body without side effects after the healing period^[^^59^^]^. Higher degradation rate of OAlg can be related to the lower Mw of OAlg than conventional Alg^[^^60^^-^^63^^]^ and facile hydrolysis of OAlg, due to the higher rotate ability of the β-glycosidic linkage as a result of C-C breaking in the glucuronide units of Alg structure by sodium periodate in the OAlg synthesize procedure^[^^11^^,^^61^^,^^64^^]^. The degradation rate of G6S0 was significantly higher than G4S2 and G0S6 samples at all pre-set time points. Approximately 89.8 ± 2.3% and 93.9 ± 2.1% of the initial weight of G6S0 sample was degraded after 21 and 28 days, respectively. Moreover, the final degradation rate for G4S2 and G0S6 samples was calculated to be 46 ± 12% and 61.01 ± 1.23 % after 28 days, respectively. It has been reported that the complete restoration of the epidermis and dermis layers occurs four weeks after surgery^[^^65^^]^. Therefore, it seems that G4S2 and G0S6 scaffolds could provide a suitable degradation rate for skin regeneration.

***Mechanical behavior***

**Fig. 4:**
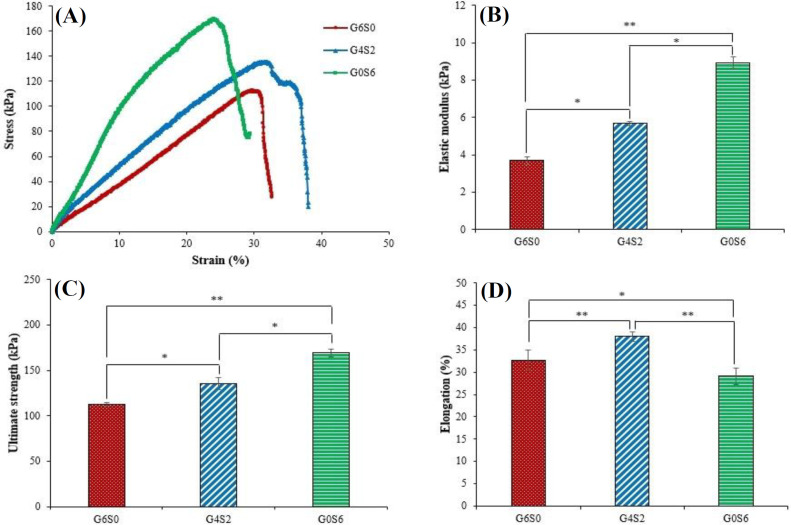
Mechanical properties of G6S0, G4S2, and G0S6 scaffolds. (A) stress-strain curves, (B) elastic modulus, (C) ultimate strength, and (D) elongation at break

The mechanical properties of the designed scaffolds should be matched with the target tissue to provide a biomimetic microenvironment^[^^66^^]^. In the bioprinting procedure, the mechanical properties affect the printability, shape fidelity, and resistance to damage during handling^[^^67^^]^. Therefore, the mechanical properties of the scaffolds were evaluated using the uniaxial tensile test, and the stress-strain curves, elastic modulus, ultimate tensile strength, and elongation at break of G6S0, G4S2, and G0S6 is shown in Figure 4. 

**Fig. 5:**
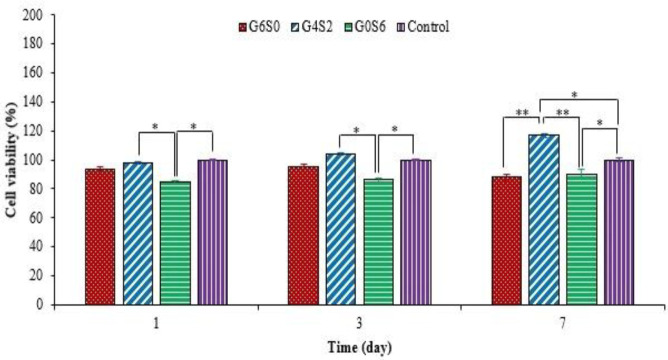
**MTT assay of G6S0, G4S2, and G0S6 scaffolds with** **encapsulated L929 fibroblasts after one, three, and seven days of treatment. All values are expressed as mean ± SD (n = 3)** **.**

According to the results, G0S6 presented the highest elastic modulus (8.93 ± 0.3 kPa) and ultimate tensile strength (169.50 ± 4.2 kPa) in comparison to the other samples. Moreover, G6S0 sample showed the lowest elastic modulus and ultimate tensile strength of 3.69 ± 0.2 kPa and 112.83 ± 2.1 kPa, respectively. The higher concentration of SF can cause the formation of β-sheets. The formation of β-sheets, in addition to covalently Schiff base crosslinking between OAlg and the SF amino groups, led to higher mechanical properties in the G0S6 scaffold^[^^43^^]^. The ultimate tensile strength and elongation of natural human skin are 2.5-16 MPa and 35-115%, respectively^[^^68^^,^^69^^]^. Therefore, the optimum bioink should provide enough elongation in addition to the regulated elastic modulus and ultimate tensile strength properties. Although G0S6 showed high elastic modulus and ultimate tensile strength, it indicated only 29.10 ± 7.9% elongation. Among the tested scaffolds, G4S2 has generally shown improved mechanical results with an elastic modulus of 5.67 ± 0.1 kPa, ultimate tensile strength of 135.50 ± 6.5 kPa, and elongation of 38.00 ± 5.1%. Thus, based on the mechanical properties, especially the proper elongation, G4S2 scaffold displayed to be potential for skin replacements.

***MTT assay***

The wound healing process is a dynamic, spontaneous, and complex process in which different cells play special functions. The interaction of the cells and biomaterial is a critical parameter in the healing process of skin injuries so that the cells can maintain their natural phenotype, proliferate and migrate^[^^70^^]^. To investigate the cell viability of OAlg-based scaffolds, the MTT assay was performed after 1, 3, and 7 days, and the results are represented in Figure 5. Although G0S6 showed the lowest cell viability compared to other samples, all samples demonstrated high cell viability. The cell viability and proliferation of G4S2 and G0S6 scaffolds increased with time, whereas there was a reduction in the cell viability of the G6S0 sample after seven days. This reduction can be related to the high degradability rate of G6S0. The cell viability of G4S2 sample was measured as 116.9 ± 1.5%, which significantly increased compared to the control sample after seven days (p < 0.05). Guo et al.^[^^11^^]^ have reported that with the extra oxidation degree of Alg (NaIO_4_ > 80%), the cell viability will be reduced due to the presence of unreacted residual sodium periodate, as well as higher produced aldehyde groups. Therefore, the partial oxidation of Alg, which is considered in this research, has an increasing effect on the cell growth and proliferation without any adverse effect on cell viability. Overall, the results showed that G4S2 had a higher capability in supporting cell viability and proliferation compared to the other tested bioink samples.

***Live/dead staining***

**Fig. 6:**
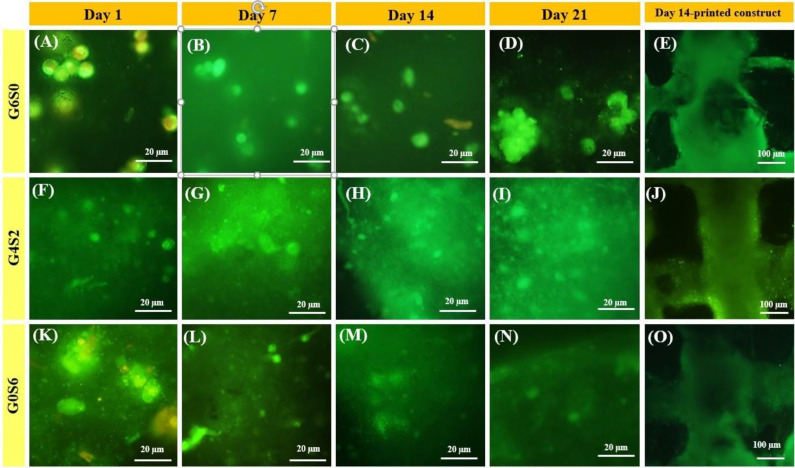
Live/dead (AO/PI) staining images of cell-laden OAlg scaffolds. G6S0 (A, B, C, and D), G4S2 (F, G, H, and I), and G0S6 (K, L, M, and N) at 1, 7, 14, and 21 days, respectively. The staining images of printed G6S0, G4S2, and G0S6 scaffolds after 21 days (E, J, and O), respectively

Figure 6 shows the fluorescent images of AO/PI stained L929 fibroblast cells encapsulated in the printed G6S0, G4S2, and G0S6 scaffolds after 1, 7, 14, and 21 days. The studies of cell behavior of bone and skin cells over 21 days have already been mentioned as long-term cell studies in previous reports^[^^71^^,^^72^^]^. Therefore, in current study, the cell viability of the encapsulated cells was investigated after 21 days. The live/dead staining images indicated the well and uniform distribution of the cells in all around the scaffolds. The green color of the cells confirmed their viability even after long-term encapsulation (21 days). The round morphology of most of the cells revealed low attachment and low interactions between bioinks and cells^[^^5^^]^. According to the cell viability measurements, G4S2 scaffold showed the highest cell viability at all the defined time points, with 93.7 ± 5.5%, 95.6 ± 4.7%, 97.2 ± 3.7%, and 98.6±2.94% cell viability at 1, 7, 14, and 21^th^ day, respectively. The G6S0 sample represented lower cell viability than G4S2 sample, which may be due to the higher viscosity of the G6S0 scaffold or its higher degradation rate compared to G4S2; therefore, the cells will not have the necessary time to grow up and secrete their own extracellular matrix ^[^^11^^,^^73^^]^. On the other hand, G0S6 scaffold showed the lowest cell viability at all interval times, likely due to the absence of GL, which could have provided more cell adhesion sites. As a result, the cell viability achievement demonstrated the high capability of the well-formulated bioinks for fibroblast cell viability with regulated viscosity, which reduced stresses on cells during the 3D printing procedure. Therefore, owing to the elimination of chemical and toxic crosslinkers in the composition of prepared bioinks, the percentage of cell viability exhibited a significant increment. The G4S2 scaffold showed the highest cell viability ratio, indicating its capability as a cell-laden bioink for 3D bioprinting applications. 

## Conclusion

In this study, OAlg-GL-SF bioink with an appropriate ratio of SF to GL was successfully synthesized. The 3D bioprinting was performed to fabricate 3D OAlg-GL-SF-based scaffolds and it was crosslinked via two-stage crosslinking procedures based on Schiff base formation and ionic gelation using CaCl_2_. The scaffolds were evaluated with different in vitro tests, and the results demonstrated the high capability of all prepared samples, whereas G4S2 sample containing 4% (w/v) OAlg, 4% (w/v) GL, and 2% (w/v) SF showed the optimum characteristics with improved printing accuracy, wettability, swelling, and degradability, as well as mechanical and cellular viability. Altogether, the cell-laden 3D bioprinted scaffold made of OAlg, GL, and SF could be considered as a potential candidate for skin tissue replacement; however, more in vitro and in vivo studies are needed to evaluate its biocompatibility and efficacy.

## DECLARATIONS

### Acknowledgments

The authors would like to thank the Stem Cell Engineering Research laboratory at Sharif University of Technology (SUT) and Biomaterials laboratory at Materials and Energy Research Center (MERC) for their assistance.

### Ethical statement

This study was carried out under an approval from the Materials and Energy Research Center for research proposal (781398058). New cell lines were not derived for this study.

### Data availability

The raw data supporting the conclusions of this article are available from the authors upon reasonable request.

### Author contributions

KS: conceiving and designing the analysis, investigating, performing the analysis, and writing and revising the original draft of manuscript; AZ: supervising, conceiving and designing the analysis, funding acquisition, and revising the draft of manuscript. SM: and TR: advisoring the project, conceiving and designing the analysis, and studying, editing, and revising the manuscript. All authors have read and approved the final version of the manuscript. 

### Conflict of interest

None declared.

### Funding/support

This study received no specific fund from any agency in the public, commercial, or not-for-profit sectors.
